# Electrochemotherapy in pediatrics: a systematic review

**DOI:** 10.1007/s00330-026-12554-z

**Published:** 2026-04-22

**Authors:** M. A. Formstone, J. J. Neville, A. N. Smith, A. C. Sedgwick, I. Diez-Perez, P. A. Patel, J. Anderson, S. Giuliani

**Affiliations:** 1 Faculty of Medicine, University of Southampton, Southampton, UK; 2 Cancer Section, Developmental Biology and Cancer Programme, UCL Great Ormond Street Institute of Child Health, London, UK; 3 Department of Specialist Neonatal and Paediatric Surgery, Great Ormond Street Hospital for Children NHS Trust, London, UK; 4 Department of Chemistry, King’s College London, London, UK; 5 Interventional Radiology, Radiology Department, Great Ormond Street Hospital for Children NHS Trust, London, UK; 6 Department of Paediatric Oncology, Great Ormond Street Hospital for Children NHS Trust, London, UK

**Keywords:** Pediatrics, Interventional, Electrochemotherapy, Vascular, Oncology

## Abstract

**Objectives:**

To systematically review the available evidence on electrochemotherapy (ECT) in pediatric patients, focusing on indications, treatment parameters, efficacy, and safety.

**Materials and methods:**

A systematic review was conducted in accordance with PRISMA guidelines and registered with PROSPERO (CRD42024575588). MEDLINE, Embase, and Cochrane Library databases were searched. Studies reporting the use of ECT in patients ≤ 18 years were included. Data extraction covered patient demographics, pathologies, electroporation parameters, agents used, and outcomes. Risk of bias was assessed using ROBINS-I.

**Results:**

Out of 1579 screened studies, 15 met the inclusion criteria, reporting at least 127 pediatric patients. Age was provided for 98 patients (pooled mean 8.5 years, range 0–17) and sex for 108 (58 female, 50 male). Two studies described tumors; the remainder reported vascular anomalies (VAs). Reversible electroporation with intralesional or intravenous bleomycin was the most common protocol. Complete response rates for tumors were 97–100%. Pooled volume reductions in VAs ranged from 52% to 100%. Most complications were minor, though serious events occurred, including sciatic nerve injury, disseminated intravascular coagulation, and airway compromise. Methodological quality was low, with small sample sizes and inconsistent reporting of multiple parameters.

**Conclusion:**

ECT is in the early stages of development but shows promising efficacy as a treatment for tumors and VAs in children. Preliminary data suggest favorable responses in children, although direct comparison with adults is limited. Further research is essential to establish standardized treatment guidelines, optimize safety, and define the role of ECT within pediatric oncology and interventional radiology. A minimum reporting dataset is proposed.

**Key Points:**

***Question*** *Explain the unmet need/clinical problem your study addresses. What is the current evidence regarding indications, efficacy, safety, and treatment parameters of ECT in pediatric patients?*

***Findings*** *Fifteen studies, including 127 children, report high response rates for tumors and VAs, but evidence is limited by small cohorts and heterogeneous reporting*.

***Clinical relevance*** *ECT shows promise as a minimally invasive treatment for selected pediatric tumors and VAs, but standardized protocols and large prospective studies are required before broader clinical adoption in pediatric oncology and interventional radiology*.

**Graphical Abstract:**

**Figure:**
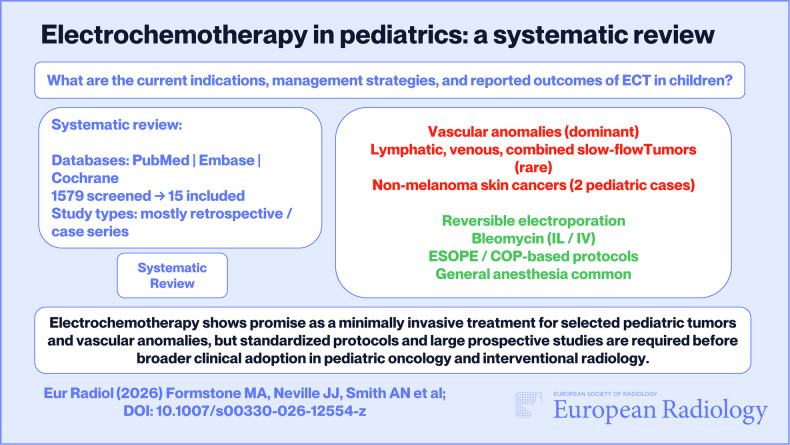

## Introduction

Electrochemotherapy (ECT) combines chemotherapeutic agents with electroporation to enhance intracellular drug delivery. Short, high-intensity electric pulses transiently increase cell membrane permeability [1], allowing otherwise poorly permeant cytotoxic drugs to enter cells at substantially increased concentrations while limiting systemic exposure. Reversible electroporation (RE) permits membrane resealing after treatment [2], whereas irreversible electroporation (IRE) uses higher-intensity pulses that permanently disrupt membrane integrity, resulting in direct cell death [3, 4].

Bleomycin is the most used chemotherapeutic agent in ECT, with electroporation markedly enhancing its intracellular uptake [5]. Cisplatin is also used [6], and preclinical studies demonstrate that electroporation can increase bleomycin cytotoxicity by several hundred-fold, consistent with manufacturer descriptions, while cisplatin shows more modest, tens-fold potentiation [7, 8]. Bleomycin remains preferred for most cutaneous lesions, whereas cisplatin may be considered in HPV-positive tumors or when bleomycin is contraindicated [9].

ECT has been widely studied in adult oncology, demonstrating high response rates across cutaneous, subcutaneous, and selected visceral tumors [10–13]. A randomized controlled trial (RCT) showed ECT with intralesional bleomycin to be equivalent to surgery for basal cell carcinoma (BCC) at five-year follow-up [14]. Further phase I/II studies have shown feasibility and safety in the treatment of pancreatic adenocarcinoma and colorectal liver metastases [15, 16]. The European Standard Operating Procedures of Electrochemotherapy (ESOPE) have standardized treatment protocols [17], facilitating broader clinical adoption, including applications in spinal metastases where emerging evidence demonstrates pain control, local tumor control, and acceptable safety [18].

Beyond oncology, ECT—often termed electrosclerotherapy (EST) when used to treat vascular anomalies (VAs)—has shown promise in both vascular malformations and vascular tumors [19, 20]. Despite these advances, ECT remains underused in pediatrics owing to limited data, differences in tissue and vascular biology, and concerns regarding long-term effects [21]. Nevertheless, emerging reports suggest that ECT may offer a valuable treatment option for refractory or inoperable pediatric tumors and VAs. This systematic review aims to summarize current indications, treatment approaches, and reported outcomes of ECT in children.

## Methodology

This systematic review was conducted in accordance with the Preferred Reporting Items for Systematic Reviews and Meta-analyses (PRISMA) guidelines. The protocol for the review was pre-registered on PROSPERO (CRD42024575588). For further methodological details, see Supplementary Materials. As this study is a systematic review of published literature, institutional review board approval and patient consent were not required.

### Literature search and study selection

A comprehensive literature search was conducted using MEDLINE via PubMed, Embase, and the Cochrane Library databases using terms relevant to ECT (Table S1). Databases were searched from inception to 26th November 2025. Duplicates were removed, and each abstract was screened independently for full text retrieval by MAF and JJN. Full texts were reviewed for inclusion in a similar manner, with any disagreements resolved by a third author (PAP). Studies were included if they described any use of ECT in children (aged ≤ 18 years). Studies of combined children and adult cohorts were included if sufficient data were available regarding management and outcomes in children.

### Data extraction and bias assessment

Data were extracted from eligible studies, including baseline characteristics and reported outcomes. Where reported data were incomplete, the study authors were contacted for further information. The quality and risk of bias of each study were assessed using the Risk Of Bias In Non-randomized Studies—of Interventions (ROBINS-I) tool [22]. Risk of bias plots were generated using the robvis tool [23]. No studies were excluded based on the risk of bias assessment.

### Outcomes

The primary outcome was to define the pathologies currently treated by ECT in children. Secondary outcomes were the chemotherapy agents and electroporation equipment used, the ECT parameters and procedural details, post-ECT outcomes, complications from ECT, and long-term follow-up.

### Meta-analysis

Given the anticipated heterogeneity in study design, outcome definitions, and reporting practices, this review was planned as a descriptive systematic review. A quantitative meta-analysis was considered only if multiple studies reported comparable outcome measures using consistent definitions and pediatric-specific data.

### Bleomycin dosing and unit standardization

Bleomycin dosing was reported inconsistently across studies, using milligrams (mg), International Units (IU), mg/kg, or total per-session dose, and at times without stating the drug concentration. As 1 mg potency corresponds to approximately 1000 IU, but this does not necessarily equal 1 mg weight, IU is considered the most standardized measure of bleomycin activity [24]. For this review, all dosing information is reported exactly as presented in the original studies, without attempting conversion between mg and IU, to avoid introducing error.

## Results

### Study selection

**Fig. 1:**
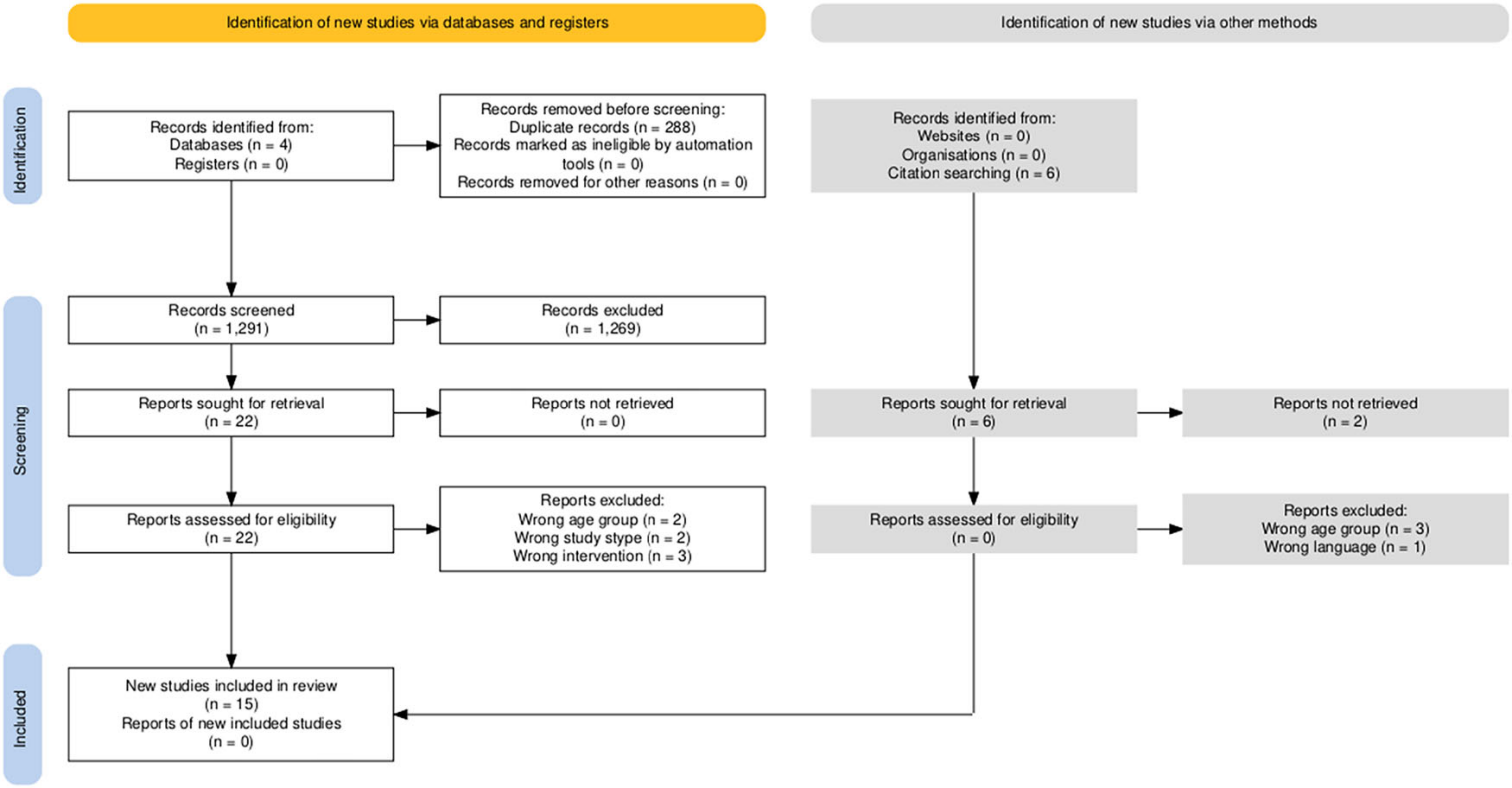
PRISMA flow diagram illustrating the selection process for studies included in the systematic review. The diagram outlines the number of records identified, screened, excluded, and included, along with reasons for exclusion at each stage (https://www.prisma-statement.org/prisma-2020-flow-diagram)

A total of 1579 studies were identified through the database search, with an additional six papers found through reference screening (Fig. 1). After removing duplicates and screening abstracts, 28 full-text articles were assessed for eligibility. Fifteen articles met the inclusion criteria [25–39], comprising three case reports [25, 26, 32], one prospective cohort [27], two prospective case series [28, 31], five retrospective cohorts [29, 33–35, 38], three retrospective case series [30, 37, 39], and one retrospective multicenter study [36].

The studies included in this review involved contributions from dermatologists [25, 30], pediatric surgeons [28, 29], plastic surgeons [26, 31], radiologists [27, 32–36, 39], spinal surgeons [37], and ENT surgeons [38]. Patients from at least 10 studies underwent surgical intervention prior to or post-ECT [26, 27, 29, 30, 32–34, 36, 38, 39].

### Patients

Nine of 15 studies included both adults and children [30, 31, 33–39], and six focused exclusively on pediatric populations [25–29, 32]. In total, 457 adults and children were studied across the included papers (Table 1). Twelve studies explicitly reported the number of pediatric patients, totaling 127. Age was reported for 98 patients (pooled mean 8.5 years, range 0–17), and sex for 108 (58 female, 50 male). Study locations included Hungary (*n* = 3), Germany (*n* = 9), Italy (*n* = 2), and the United Kingdom (*n* = 1).

**Table 1:** Table outlining study demographics

Study (location)	Total cohort size	Pediatric patient cohort size (%)	Overall age in years (median/mean) (range)	Overall/pediatric male: female ratio (male%)	Study design
Baltas et al (Hungary) [25]	1	1 (100)	11 y	0:1 (0)	Case report
Dalmady et al (Hungary) [26]	1	1 (100)	infant^*^	1:0 (100)	Case report
Goldann et al (Germany)^**^ [27]	30 → 24	30 (100) → 24 (100)	Mean 9.5 y (1–17)	10:14 (41.7)	Prospective cohort
Guida et al [28] (Italy)	12	12 (100)	Median 6.8 y (< 1–15; IQR 3.8–10.7)	4:8 (33.3)	Prospective case series
Haehl et al [29] (Germany)^***^	45	45 (100)	Median 8.0 y (0–17)	23:22 (51.1)	Retrospective cohort
Kis et al (Hungary) [30]	12	1 (8.33)	Mean 61.6 y (11–86)	7:5 (58.33)/0:1 (0)	Retrospective case series
Kostusiak et al (UK) [31]	30	4 (13.33)	Mean 42 y (6–75)	13:17 (43.33)/n/a	Prospective case series
Loeser et al (Germany) [32]	1	1 (100)	2 y	1:0 (100)	Case report
Loeser et al [33] (Germany)	12	8 (66.67)	Mean 14.75 y (10d–35)	7:5 (58.3)/6:2 (75)	Retrospective cohort
Obereisenbuchner et al [34]—LMs (Germany)	20	13 (65)	Median 12.5 y (2–53)	10:10 (50.0)/n/a	Retrospective cohort
Obereisenbuchner et al [35]—AVMs (Germany)	21	5 (23.8)	Median 33 y (0.5–53)	9:12 (42.9)/2:3 (40)	Retrospective cohort
Schmidt et al^****^ (Germany) [36]	233	n/a	Median 13 y (0–71)	118:115 (50.64)/n/a	Retrospective multicenter observational study
Tedesco et al (Italy) [37]	2	1 (50)	16 y; 50 y	0:2 (0)/0:1 (0)	Retrospective case series
Vielsmeier et al [38] (Germany)	20	n/a	Median 39 y (4–78)	7:13 (35.0)/n/a	Retrospective cohort
Wohlgemuth et al (Germany) [39]	17	10 (58.8)	Mean 20.8 y (10–31)	8:9 (47.06)/2:6 (25)	Retrospective case series

*UK* United Kingdom, *USA* United States of America

^*^ First ECT treatment given when the child was a few months old

^**^ Six children were lost to follow-up; the mean and sex ratio were based on 24 patients

^***^ Text reports median 6.5 years; table reports median 8 years; the table value was extracted for consistency

^****^ Did not specify the exact number of children in the study population

### Pathologies treated with ECT in children

The primary outcome was identifying the types of pediatric pathologies treated with ECT in the literature (Table 2), including lesion site and size when documented. Studies were categorized into two groups: benign and malignant tumors [25, 30], and VAs (including vascular tumors, such as hemangiomas) [26–29, 31–39]. Where possible, adult pathologies were excluded.

**Table 2:** Pediatric-specific pathologies treated by ECT

Study	No. of children	General pathology	Specific pathology	Described sites of lesion(s)	Size of lesions
Baltas et al [25]	1	Benign and malignant tumors	Xeroderma pigmentosum; NMSCs; SCC; KA lesion (progressive)	The majority localized to the head and neck (82%)	Mean diameter = 0.85 cm (range 0.3–3.6 cm)
Dalmady et al [26]	1	VAs	Lymphatic malformation	Neck	72.32 cm^3^
Goldann et al [27]	24	VAs	Vascular malformations	Two figures note knee and thigh lesions	Median volume = 232 cm^3^ (range: 1.69–9814.12 cm^3^)
Guida et al [28]	12	VAs	Vascular malformations	Buttock, lower limb, upper limb, foot, neck, tongue, knee, lumbosacral	n/a
Haehl et al [29]	45	VAs	Vascular malformations	Head, neck, lower extremity, upper extremity, trunk, buttocks	n/a
Kis et al [30]	1	Malignant tumors	Locally advanced/recurrent periocular BCCs	Upper and lower eyelids, both canthal regions, nose, perioral skin, frontotemporal area	Periocular tumors: 6 × 8; and 12 × 5 mm
Kostusiak et al [31]	4	VAs	Vascular malformations	n/a	n/a
Loeser et al [32]	1	VAs	Microcystic lymphocytic malformation	Tongue (bilateral, with cervical extension)	Dimensions not specified. See Fig.1 in Loeser et al
Loeser et al [33]	8	VAs	Vascular malformations	Tongue, axilla, thorax wall, face, neck, temple, orbita, groin, penis root, scrotum, left arm	Mean volume = 88.76 cm^3^ (range: 0.38–541.5 cm^3^)
Obereisenbuchner et al [34] LMs	13	VAs	Lymphatic/lymphatic-dominant lympho-venous malformations	Head, neck, lower extremity, thorax, pelvis, upper extremity, abdomen	Mean maximum volume = 793 cm^3^ (IQR 155–2199 cm^3^)
Obereisenbuchner et al [35] AVMs	5	VAs	Arteriovenous malformations	Lip, enoral/oral cavity, periorbital, extremities, trunk	n/a
Schmidt et al [36]	n/a	VAs	Vascular malformations	Head, neck, lower extremity, upper extremity, trunk, buttocks	n/a
Tedesco et al [37]	1	VAs	Aggressive spinal hemangiomas	T12	Dimensions not specified. See Fig. 3 in Tedesco et al
Vielsmeier et al [38]	n/a	VAs	Vascular malformations (of the upper aerodigestive tract)	Laryngeal and hypopharyngeal mucosa, tongue, and oral cavity	n/a
Wohlgemuth et al [39]	8	VAs	Venous malformations	Thigh, knee, foot, flank, gluteal area, calf	Ranged from 8.14 cm^3^ to 334.80 cm^3^

*NMSC* non-melanoma skin cancer, *SCC* squamous cell carcinoma, *KA* keratoacanthoma, *BCC* basal cell carcinoma

#### Benign and malignant tumors

Only two pediatric patients with tumors and lesions are reported in the literature (Table 2) [25, 30]. Both cases involved female patients aged 11-years-old. Tumor types included multiple NMSCs ((non-melanoma skin cancers) at least 23, with the clinical appearance of BCCs), one squamous cell carcinoma (SCC), one KA, and two periorbital BCCs. Tumor sizes varied, with Baltas et al reporting a mean lesion diameter of 0.85 cm (0.3–3.6) and Kis et al describing two periocular tumors of 6 × 8 and 12 × 5 mm.

#### VAs

One-hundred and twenty-five cases involving VAs are reported in the literature (Table 2) [26–29, 31–39]. Schmidt et al and Vielsmeier et al also studied pediatric patients but did not specify exact numbers [36, 38]. Not all studies provided specific data for lesion type, site, or size of lesions. When they were reported, pathologies included various vascular malformations, and one spinal hemangioma. Lesion sites and sizes varied significantly as described in Table 2, ranging from a few square centimeters to almost 10,000 cm^3^.

### ECT treatment details and outcomes

#### Benign and malignant tumors

Bleomycin was the chemotherapeutic agent in both tumor ECT studies, delivered intravenously in Baltas et al and in most cases in Kis et al, with both groups adhering to contemporary ESOPE guidance and using the Cliniporator™ platform. Electrical parameters varied considerably between studies (Table 3), reflecting differences in electrode type and tumor anatomy. Baltas et al used type II and type III electrodes with standard ESOPE pulse regimens [15, 18], whereas Kis et al applied electrode-specific settings, including lower-voltage hexagonal arrays and current-controlled row-needle electrodes for their pediatric case.

**Table 3:** Summary of pediatric-specific electroporation type, ECT parameters, route, type, and dose of chemotherapy

Study	No. of children	Reported electrodes	Electrical parameters (pulses, V/cm, duration, Hz, A, cycles)	Route, type, dose, and timing of chemotherapy
Baltas et al [25]	1	Parallel arrays or plate electrodes (type II); hexagonal array needle electrodes (type III)	Type II: 8 pulses, 1000 V/cm, 100 ms, 1 or 5000 Hz. Type III: 96 pulses (8/pair), 1000 V/cm, 100 ms, 5000 Hz	IV bleomycin 15 mg/m^2^, 8–28 min pre-electroporation (pharmacokinetic peak).
Dalmady et al [26]	1	Row needle electrodes	Eight consecutive pulses per application, 1000 V/cm, 100 μs per pulse, 5.27 A or 5.55 A (2–14 A), 68 and 74 applications	IL bleomycin 0.5 mg/kg (equivalent to 8100 IU total), immediate electroporation.
Goldann et al [27]	24	Linear, hexagonal, finger, or needle electrodes	Mean number of cycles per session = 26.19 (SD 31.11)	IV or IL bleomycin. IV: pulses 8 min after infusion. IL: pulses 1–3 min after injection. Mean 3.86 mg/session. Max 1 mg/5 kg, cumulative < 5 mg/kg.
Guida et al [28]	12	Adjustable-length electrodes	Authors describe applying “one or more electrical pulses” after bleomycin administration	IL bleomycin 0.5 mg/kg → pulses immediately. IV bleomycin 0.3 mg/kg → pulses 10 min after infusion.
Haehl et al [29]	45	Finger and hexagonal electrodes	100 µs; ≤ 3000 V; ≤ 50 A; median 20 cycles (4–141)	IL bleomycin 64/68 sessions (mean 5.9 mg). IV 2/68 (3.4 mg). Combined 2/68 (6.5 mg). Max 0.2 mg/kg/session, cumulative < 1 mg/kg. IL = immediate electroporation, IV = 8 min pre-electroporation.
Kis et al [30]	1	Row needle electrodes	Eight pulses, 1000 V/cm, 100 ms, 5 kHz, 2-3 A	IV bleomycin 15,000 IU/m^2^, 8 min pre-electroporation.
Kostusiak et al [31]	4	Handheld finger or linear electrode	n/a	IL bleomycin, lesion-size–based dose + lidocaine; immediate electroporation.
Loeser et al [32]	1	Finger electrode	n/a	IL bleomycin 1–2 mg/session, immediate electroporation.
Loeser et al [33]	8	Finger, triangularly arranged VGD needle, hexagonal adjustable, and linear adjustable electrodes	100 µs; ≤ 3000 V; ≤50 A; mean 24 pulse series (2–82)	IL bleomycin 3.65 mg (0.5–15 mg), median cumulative 6.38 mg. IV bleomycin (0.17–0.25 mg/kg). IL = immediate electroporation, IV = “without delay.”
Obereisenbuchner et al [34]—LMs	13	Finger and hexagonal electrodes	100 µs; ≤ 3000 V; ≤ 50 A; median 26 cycles (6–110)	IL bleomycin, median 7 mg (2–15 mg); cumulative < 1 mg/kg; immediate electroporation.
Obereisenbuchner et al [35] –AVMs	5	Hexagonal, finger, and freely positionable needle electrodes	100 µs; ≤ 3000 V; ≤ 50 A; mean 20.6 cycles (4–70). Needle spacing 0.5–3 cm	BEST: IV bleomycin → pulses 8 min after infusion. BEET: IA bleomycin → pulses 1 min after infusion. Max 0.2 mg/kg/session, cumulative < 1 mg/kg.
Schmidt et al [36]	n/a	Hexagonal, finger, and freely positionable needle electrodes	100 µs; ≤ 3000 V; ≤ 50 A	IL bleomycin in 94.8% of sessions (median 3 mg (0.5–25 mg)); IV bleomycin in 9.8% (median 5 mg (1–15 mg)); combined 3.1% (median 6 mg, (4–21 mg)). IL = immediate; IV = 8 min; max 0.2 mg/kg/session, cumulative < 1 mg/kg.
Tedesco et al [37]	1	Trocar-type electrodes	n/a	IV bleomycin 15 mg/m², 8 min pre-electroporation.
Vielsmeier et al [38]	n/a	Finger and “Stinger” electrodes	100 µs pulses; ≤ 3000 V/cm; ≤ 50 A; mean 12.7 cycles	IL bleomycin 28/29 sessions (mean 5.7 mg). IV 1/29 (7 mg). IL = immediate; IV = 8 min; max 0.2 mg/kg/session, cumulative < 1 mg/kg.
Wohlgemuth et al [39]	8	Needle electrodes (various configurations, see Fig. 1 in Wohlgemuth et al)	Consecutive pulses over 10 min, 1000 V/cm, 100 μs	IL bleomycin 0.25 mg/mL (max 1 mg/kg/session or 1 mL/cm³ lesion), immediate.

*RE* reversible electroporation

Note that ‘cycle’, ‘application’, and ‘series’ appear to be synonymous

Despite these methodological differences, both studies reported excellent tumor control (Table 4). Baltas et al achieved a 97% complete response rate without tumor recurrence during follow-up, while Kis et al reported 100% histologically confirmed clearance of periocular lesions. Procedure-related morbidity was limited, although Kis et al noted postoperative ectropion in three cases, including the pediatric patient, requiring surgical correction.

**Table 4:** Summary of pediatric-specific outcomes and complications

Study	No. of children	Outcomes (e.g., CR)	Complications
Baltas et al [25]	1	CR in 97% of tumors	Mild, transient sore muscles, local erythema/edema, and temporary needle-mark discoloration were observed. One lesion showed disease progression.
Dalmady et al [26]	1	Absolute volume change: 72.32 → 48.09 cm³ (33.5%); 63% growth-corrected target volume reduction	Prominent postoperative edema occurred but resolved without intervention. Two episodes of erysipelas at 1 and 2 months post-treatment, both resolving with antibiotics. Mild skin scars and slight hyperpigmentation persisted at follow-up.
Goldann et al [27]	24	Median baseline volume was 232 cm^3^ (1.69–9814.12), decreasing to 73.29 cm^3^ after the first session (38.7%) and 41 cm³ at final follow-up, corresponding to a mean relative reduction of 59.4%. Symptomatically, 2/24 became asymptomatic, 18/24 improved, and 4/24 remained unchanged.	Transient skin hyperpigmentation in 6/24, localized inflammation in 3/24, temporary knee-motion restriction in 1/24, and lymphorrhea after a second session in 1/24; all resolved without sequelae.
Guida et al [28]	12	Clinical improvement in all cases; 6/8 patients with ≥ 5 months follow-up achieved complete resolution; 2/8 showed marked ultrasonographic reduction; 4 patients remained under treatment.	Two patients (17%) developed transient skin dyschromia; one (8%) had a self-limiting tongue hematoma. All resolved spontaneously without sequelae.
Haehl et al [29]	45	Major symptomatic improvement: 97.8% → 35.6% moderate/severe; mobility improved in 34.1%; symptom-free in 24.4%; esthetics improved/perfect in 80.5%; social participation improved/perfect in 85.4%; pain VAS 2.0 → 0.0.	Complications occurred after 10/68 sessions (14.7%). Pes equinus deformity developed after all calf-muscle treatments (5/68), requiring physiotherapy in three cases and Achilles tendon lengthening in two. Post-interventional bleeding occurred twice (2/68), including one life-threatening hemorrhage requiring embolization and ICU care. Wound infections occurred in two sessions (2/68), and one child developed a transient wrist flexion contracture (1/68).
Kis et al [30]	1	CR	Ectropion caused by post-ECT scar requiring surgical correction.
Kostusiak et al [31]	4	n/a^*^	Minor, self-limiting complications were common: swelling (*n* = 6), infection (*n* = 3), pain (*n* = 2), and bleeding (*n* = 2), with additional complaints of weeping, drooling, crusting, and ulceration.^*^
Loeser et al [32]	1	Significant lesion size reduction with improved function	n/c
Loeser et al [33]	8	Volumetry available for 17 pediatric lesions. Mean relative volume reduction 57.0%; median 52.6% (range 17–100%). 11/17 showed ≥ 50% reduction, 6/17 ≥ 70%, and 2/17 achieved complete (100%) radiological resolution. All patients had symptomatic improvement.	Adverse events: reversible tongue swelling (*n* = 1); severe swelling with limited eye opening (*n* = 1); reversible functional impairment of the shoulder (*n* = 1). Complications: temporary injection site discoloration (*n* = 1).
Obereisenbuchner et al [34]—LMs	13	MRI volumetry available in 14/20 patients showed a reduction in mean maximum volume from 793 cm³ (IQR 155–2199) to 548 cm³ (IQR 71–1059) (*p* = 0.048). MRI was omitted in six pediatric patients to avoid anesthesia. Complete subjective response in 7/20 (35.0%).^*^	Total complication rate (CIRSE grade 3–4) was 11.1%.^*^
Obereisenbuchner et al [35]—AVMs	5	MRI available in 16/21 patients: complete devascularization 1/16 (6.3%), substantial 6/16 (37.5%), partial 6/16 (37.5%), progression 3/16 (18.8%). Clinical outcomes: 4/21 (19.0%) symptom-free, 13/21 (61.9%) improved, 2/21 unchanged, 2/21 progressed. Pediatric subgroup (≤ 4 y: *n* = 4; 13 y: *n* = 1): 4/5 showed partial or complete improvement; 1/5 progressed.	Postprocedural complications occurred after 7/31 interventions (22.6%): excessive swelling (*n* = 4), postprocedural hematoma with delayed wound healing requiring split-skin grafting (*n* = 1), persistent scarring not requiring surgery (*n* = 1), and transient jaw pain after BEET (*n* = 1). Local skin discoloration was frequent (*n* = 14), fading over time.^*^
Schmidt et al [36]	n/a	Children (< 16 y; *n* = 62): mobility improved/symptom-free 71%; esthetics improved/perfect 75.8%; pain VAS 3 → 0; pain reduced/regressed 56.5%; all significantly better vs adults.^*^	Total complication rate 10.2% (33/325), including 29/352 (8.9%) major complications. See Table 5.^*^
Tedesco et al [37]	1	Stable lesion, occasional pain; planned repeat ECT	n/c
Vielsmeier et al [38]	n/a	Significant reduction 55%, partial 40%, stable 5%; strongest responses in tongue and oral cavity + tongue, weakest in laryngeal/hypopharyngeal lesions. *	Postprocedural swelling was common (*n* = 14/20); pain (*n* = 3), bleeding (*n* = 4), dysphagia (*n* = 6), and infection (*n* = 1) were less frequent. Severe airway events were confined to laryngeal/hypopharyngeal lesions, with dyspnea requiring tracheostomy or temporary intubation (*n* = 4) and life-threatening bleeding (*n* = 1).^*^
Wohlgemuth et al [39]	8	Pediatric subset (9 lesions in 8 patients, 10–17 y): mean volume reduction 88.4%, median 97.7% (range 52–100%); 2/9 lesions fully resolved on MRI. All children improved clinically or were asymptomatic.	Temporary skin hyperpigmentation (*n* = 1); mild pain, swelling (*n* = 2); small skin lesion (*n* = 2); Skin discoloration (*n* = 1)

*VAS* visual analog score; *n/c* no complications

^*^ Pediatric-specific outcomes not available

#### VAs

Across studies that reported electroporation parameters, pulse duration was consistently 100 µs, with devices capable of delivering voltages up to 3000 V and currents up to 50 A (Table 3). The number of electroporation cycles varied widely: larger pediatric cohorts reported median or mean values between 12.7 and 26 cycles per session, with upper ranges extending to 70–141 cycles. Importantly, a cycle does not represent repeated power application at the same location (which could result in an irreversible effect), but rather sequential applications with changing needle positions across the lesion to ensure coverage of its full extent; therefore, the number of cycles is lesion-size dependent. Case reports described more granular parameters, including 8 pulses per application at 1000 V/cm with 68–74 applications per session [26]. Several studies did not provide cycle counts, and some described electroporation qualitatively (e.g., “one or more pulses” or “pulses delivered over 10 min”) [28, 39]. Specific current measurements were reported only in single-patient studies, with values ranging from 2 to 14 A [26, 30].

Bleomycin was the only chemotherapeutic agent used (Table 3), delivered IL or IV, and in one pediatric facial AVM case via intra-arterial Bleomycin ElectroEmboloTherapy (BEET)[F]. Most studies adhered to up-to-date COP-based (current operating procedure) protocols [17, 24, 40]. Bleomycin dosing units were reported inconsistently across studies (mg, mg/kg, IU, etc.). IL administration was the most frequently used route, with fewer cases receiving IV or combined IL/IV dosing.

Electrode choice, when explained, depended on lesion depth and anatomical constraints, and across studies included finger, hexagonal, linear, row, adjustable-length, freely positionable needle, and trocar-type configurations. The Cliniporator™ (IGEA Ltd.) device was universally used.

General anesthesia (GA) was the predominant modality where pediatric-specific data were provided, although some series employed regional anesthesia or procedural sedation [28, 31], and one pediatric case in Tedesco et al was treated under ‘deep sedation with spontaneous breathing’ [37].

The type of outcome reported varied across studies (Table 4). Some studies used volume reduction (e.g., relative or absolute); others focused on symptomatic improvement via patient questionnaires, or clinical observation for resolution. Not all studies provided pediatric-specific data that was extractable. Complications were overall low and varied depending on anatomical location and specific pathology. Seven studies made use of the CIRSE classification to report complications [41].

### Bias assessment

Given the inclusion of small case reports and case series, some studies were assessed as having a serious risk of bias (Fig. S1). Nine studies were judged to have a moderate risk of bias, reflecting more structured study designs with predefined eligibility criteria, clearer outcome definitions, and lower risk across multiple ROBINS-I domains, including outcome measurement and missing data.

## Discussion

This systematic review provides the first comprehensive synthesis of the published evidence on ECT in pediatric patients. We identified 15 studies reporting outcomes in at least 127 children, with additional pediatric cases included in mixed-age cohorts where pediatric-specific data could not be reliably extracted [36, 38]. Across these studies, ECT was used predominantly for VAs, with only very limited experience in pediatric tumors. While reported outcomes are generally encouraging, the evidence base remains constrained by small sample sizes, retrospective designs, and substantial heterogeneity in technique and outcome reporting.

### Efficacy and safety in pediatric tumors

Experience with ECT in pediatric tumors is extremely limited, with only two small case-based reports identified [25, 30]. Despite this, both studies reported excellent local tumor control with acceptable morbidity, suggesting that pediatric responses may be comparable to those observed in adults, where ECT is an established option for selected cutaneous and subcutaneous malignancies [10–14, 42, 43]. However, the pediatric evidence is restricted to highly selected cases with short- to intermediate-term follow-up, precluding conclusions regarding durability, generalizability, or comparative effectiveness. At present, the use of ECT for pediatric tumors should therefore be regarded as experimental and considered only in carefully selected circumstances.

### Efficacy in VAs

In contrast, the overwhelming majority of pediatric ECT experience relates to the treatment of VAs [26–29, 31–39]. Across included cohorts, meaningful volume reduction and symptomatic improvement were consistently reported for low-flow lesions, including lymphatic, venous, and combined malformations. Relative volume reductions typically ranged from approximately 50–60% [27, 33], with some series reporting near-complete or complete devascularization in selected cases [35, 39]. Importantly, several studies documented substantial improvements in pain, function, esthetics, and social participation [27, 33, 36, 38, 39], highlighting the potential clinical relevance of ECT beyond radiological response alone.

Nonetheless, responses were not uniform. More recent and larger series demonstrate that a proportion of lesions exhibit only partial response, stable disease, or progression despite repeated treatments [27, 34, 38]. This was particularly evident in extensive, anatomically complex, or mixed-flow lesions, including those involving the oral cavity and upper aerodigestive tract [35, 38]. These findings suggest that ECT is most effective for well-defined, predominantly low-flow lesions with accessible anatomy, whereas large, infiltrative, or high-risk lesions may require combined or alternative treatment strategies.

### Complications and anatomical risk

Overall, the complication profile of pediatric ECT appears acceptable but not trivial. Most reported adverse events were minor and self-limiting, including pain, swelling, transient skin changes, and superficial ulceration [26–28, 31, 33]. However, several studies reported serious complications, such as post-procedural hemorrhage requiring intervention, airway compromise in upper aerodigestive tract lesions, disseminated intravascular coagulation after treatment of extensive lymphatic malformations, and persistent neurological deficits related to nerve injury [29, 36, 38].

These complications were strongly associated with lesion location, size, and proximity to critical neurovascular structures, rather than with ECT as a technique per se [36, 38]. They underline the importance of careful patient selection, multidisciplinary planning, and close peri-procedural monitoring, particularly for lesions involving the airway or major neurovascular bundles. Long-term follow-up is essential to capture delayed functional or growth-related sequelae in children.

### Heterogeneity of techniques and outcome definitions

Interpretation of the pediatric ECT literature is substantially limited by methodological and technical heterogeneity. Across studies, there was wide variation in lesion characterization, electroporation parameters, electrode configurations, bleomycin dosing and units, routes of administration, number of treatment sessions, and anesthesia practices [26–29, 31–39]. Outcome reporting was similarly inconsistent, encompassing diverse radiological metrics, qualitative assessments, and patient- or parent-reported outcomes.

Crucially, there is no agreed-upon definition of treatment success in pediatric ECT. Only a small minority of studies pre-defined numerical thresholds for response [31], while most reported outcomes descriptively without explicit criteria for complete, partial, or failed treatment [27–30, 33–39]. As a result, outcomes are not directly comparable across studies, and any attempt at quantitative synthesis would risk producing statistically unstable or clinically misleading estimates. For this reason, a formal meta-analysis was considered but deemed inappropriate.

### Reversible vs IRE

ECT relies on RE, which transiently increases cell membrane permeability to facilitate intracellular delivery of chemotherapeutic agents such as bleomycin [7, 44, 45]. At clinically applied pulse parameters, RE primarily functions as a permeability enhancer rather than a stand-alone cytotoxic modality, with most direct cell death attributable to chemotherapy-induced mechanisms [7, 46, 47]. Although a small proportion of cells may undergo accidental cell death following RE alone under specific conditions, this is not the intended therapeutic mechanism [48, 49].

This distinction is clinically important, as IRE represents a fundamentally different ablation strategy, relying on permanent membrane disruption and direct cell death without chemotherapy [50–53]. Clarifying this difference is particularly relevant in pediatric practice, where long-term tissue effects and functional preservation are critical considerations.

### Terminology and classification of VAs

Accurate classification of VAs is essential, as natural history, treatment response, and complication risk vary markedly between lesion types. Use of the ISSVA classification facilitates meaningful comparison across studies and supports appropriate clinical interpretation [19]. Several included studies adhered to ISSVA terminology [27, 33, 36], while others used outdated or non-standard labels or failed to clearly define flow characteristics [26]. In the context of a rare and evolving intervention such as pediatric ECT, inconsistent terminology represents a significant methodological limitation and should be addressed in future research.

### Relationship to standard therapies

Standard bleomycin sclerotherapy without electroporation is already an established treatment for many pediatric VAs [54, 55], and ECT has largely been applied in refractory, extensive, or anatomically challenging cases [27, 33, 36, 38]. None of the included studies directly compared ECT with conventional sclerotherapy, surgery, or radiotherapy in children. This selection bias is important when interpreting reported efficacy, as favorable outcomes in heavily pre-treated or complex cases are encouraging but do not define the optimal position of ECT within treatment algorithms. Comparative studies are needed to clarify whether ECT offers advantages in efficacy, safety, anesthetic burden, or durability over existing standards.

### Toward standardized reporting: a minimum data set

In response to the substantial heterogeneity identified, we propose a minimum reporting dataset for pediatric ECT studies (Table 5). This pragmatic framework outlines essential elements relating to lesion characterization, electroporation parameters, bleomycin dosing, treatment logistics, outcome definitions, complication grading, and follow-up. Adoption of such a baseline standard would substantially improve interpretability, facilitate cross-study comparison, and enable future meta-analyses as the evidence base grows.

**Table 5:** A proposed minimum reporting data set, with checklist, for standardized reporting of ECT in pediatric practice

Domain	Item to report	Minimum detail required	✓
Study design and population	Study design and setting	Prospective/retrospective; single-/multicenter; study period	▯
	Eligibility criteria	Age range, pathology, prior treatments	▯
	Cohort description	Number of patients and lesions; multiple lesions per patient stated	▯
	Pediatric demographics	Age (median/mean, range) and sex; age threshold used	▯
	Mixed-age cohorts	Pediatric outcomes are reported separately	▯
Lesion characterization	Diagnosis	ISSVA classification: combined lesions specified; benign/malignant tumor	▯
	Flow type (VAs)	Low-/high-/mixed-flow; method of assessment	▯
	Anatomical location	Site, depth, and laterality if relevant	▯
	Size/volume	Baseline measurements and imaging modality	▯
	Symptom burden and indication	E.g., pain, bleeding, functional/esthetic issues, airway risk	▯
	Prior/concurrent therapies	Sclerotherapy, surgery, embolization, systemic therapy	▯
ECT procedural details	Device and electrodes	Electroporator model; electrode type/configuration	▯
	Pulse parameters	Pulses, voltage (V/cm), duration (µs), frequency (Hz/kHz), current (A), cycles/applications	▯
	Treatment coverage	Overlap/margins; use of image guidance	▯
Bleomycin/chemotherapy details	Drug and route	IL/IV/IA administration	▯
	Per-session dose	mg (and IU if used); mg/kg for pediatric dosing	▯
	Injectate details	Concentration and volume for IL dosing	▯
	Cumulative dose	Total mg and mg/kg	▯
	Drug–pulse interval	Timing between drug administration and electroporation	▯
Anesthesia and peri-procedural care	Anaesthesia modality	GA, deep sedation, regional, or local; airway plan	▯
	Safety precautions	Antibiotics, thromboprophylaxis, ICU/airway readiness	▯
	Treatment course	Number and spacing of sessions; retreatment criteria	▯
Efficacy outcomes	Primary endpoint	Pre-specified primary measure (e.g., volumetry at 6 months)	▯
	Objective response	Radiological volumetry/devascularisation with CR/PR/SD/PD definitions	▯
	Symptoms/function	Pain, bleeding, mobility, swallowing, esthetics, participation	▯
	PROMs	Patient/parent-reported measures	▯
	Follow-up time points	Fixed, pre-defined evaluation time points	▯
Safety outcomes	Complication framework	CIRSE or equivalent classification used	▯
	Complication details	Type, grade, timing, management, resolution/persistence	▯
	High-risk events	Airway compromise, neurological injury, functional deficits	▯
	Serious adverse events	Life-threatening complications/mortality	▯
Follow-up	Duration	Median follow-up; proportion with ≥ 12–24 months	▯
	Recurrence/progression	Definitions and rates of recurrence/progression	▯
	Growth/development	Effects on limb, craniofacial, or functional development	▯

### Future directions

Given the rarity and heterogeneity of pediatric cases, single-center series are likely to remain small. Multicenter prospective registries and collaborative networks are therefore crucial [20, 24, 40]. Future studies should employ standardized terminology, pre-defined outcomes, structured complication reporting, and age-specific data presentation. Comparative effectiveness studies against established therapies will be essential to define the role of ECT as a primary, adjuvant, or salvage treatment modality in pediatric oncology and interventional vascular therapy.

## Conclusion

This systematic review shows that ECT is increasingly used in pediatric practice and demonstrates encouraging efficacy, particularly for VAs. However, substantial heterogeneity in patient selection, lesion classification, treatment parameters, and outcome reporting limits comparability and precludes formal meta-analysis. Although most reported complications are minor, serious adverse events have been described and appear closely related to lesion extent and anatomical location, highlighting the importance of careful case selection and multidisciplinary management.

The current evidence base is limited by small, predominantly retrospective studies and inconsistent reporting of pediatric-specific data. Standardized terminology, outcome definitions, and procedural reporting are therefore essential to advance the field. The proposed minimum reporting dataset aims to support more robust future studies and facilitate meaningful evidence synthesis. Prospective multicenter and comparative studies will be required to define the optimal role of ECT within pediatric oncology and interventional vascular therapy.

## Supplementary information

ELECTRONIC SUPPLEMENTARY MATERIAL

## Abbreviations

BCC: Basal cell carcinoma
BEET: Bleomycin electroembolotherapy
COP: Current operating procedure
ECT: Electrochemotherapy
ESOPE: European Standard Operating Procedures of Electrochemotherapy
GA: General anesthesia
IRE: Irreversible electroporation
IU: International units
KA: Keratoacanthoma
NMSCs: Non-melanoma skin cancers
RE: Reversible electroporation
VAs: Vascular anomalies
