# Supplementary material for: Electrochemotherapy in pediatrics: a systematic review
Source: Eur Radiol. 2026 Apr 22;36(9):7278–91. doi: 10.1007/s00330-026-12554-z (PMC13451310; doi:10.1007/s00330-026-12554-z)
Supplement: Supplementary file 1 [file 330_2026_12554_MOESM1_ESM.pdf]

ELECTROCHEMOTHERAPY IN PEDIATRICS: A SYSTEMATIC REVIEW

ELECTRONIC SUPPLEMENTARY MATERIAL

Database	Search terms	Filters
PubMed, Embase, Medline	("Electroporation" OR "Electrochemotherapy" OR “Electrosclerotherapy”) AND ("Child" OR "Children" OR "Pediatrics" OR "Infant" OR "Adolescent" OR "Neonate" OR "Youth" OR "Adolescence")	None
Cochrane Library	("Electrochemotherapy" OR “Electrosclerotherapy”) AND ("Pediatrics")	None

Table S1: Summary of search terms used.

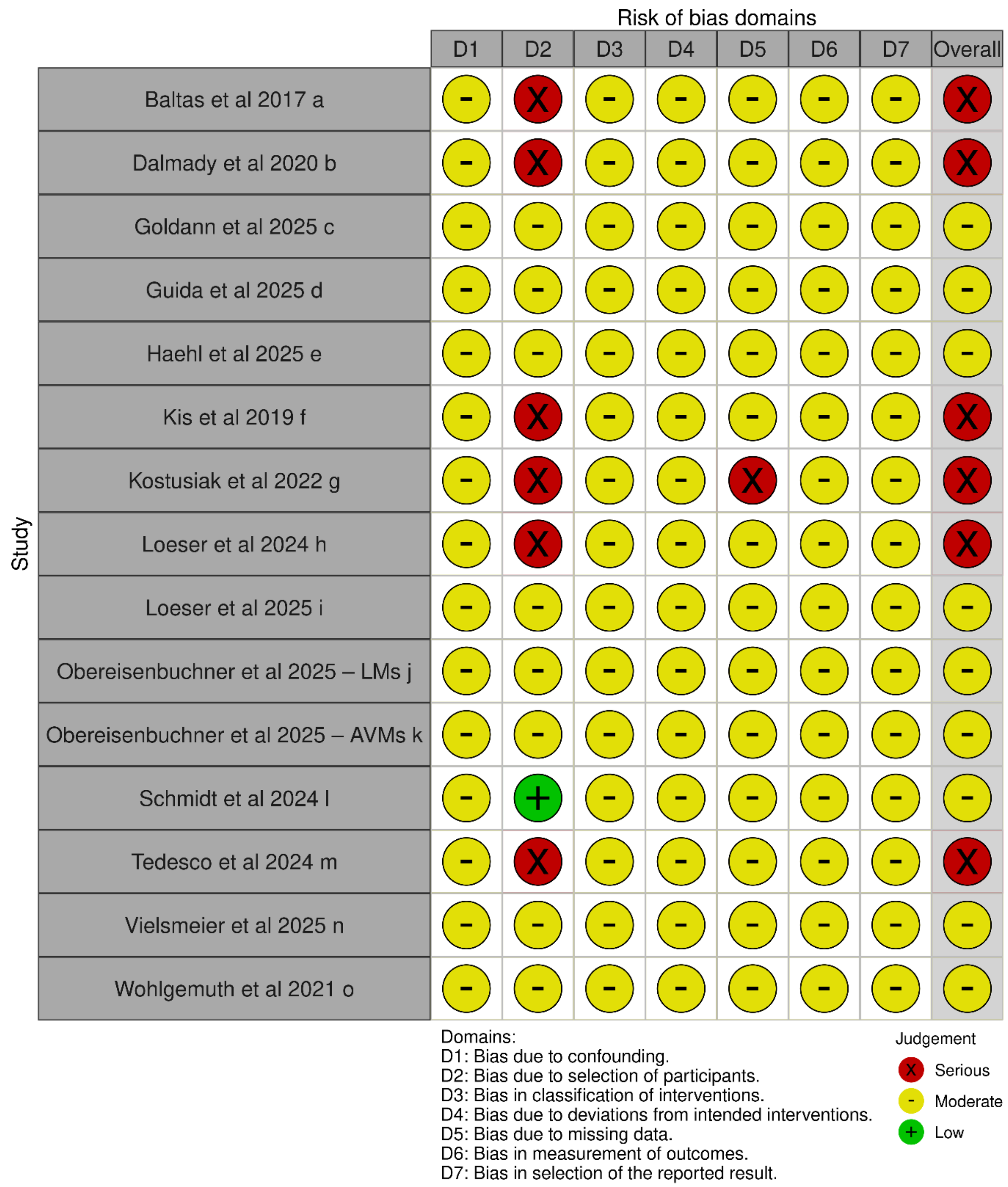

Figure S1: Risk of bias assessment using Risk Of Bias In Non-randomized Studies - of Interventions (ROBINS-I) tool[20]. (a, b, f, g, h, m) Serious risk of bias due to selection of participants due to low number of participants. (g) Serious risk of bias due to lack of extractable pediatric-specific data.
